# Novel *SMAD3* variant identified in a patient with familial aortopathy modeled using a zebrafish embryo assay

**DOI:** 10.3389/fcvm.2023.1103784

**Published:** 2023-02-28

**Authors:** Mary B. Sheppard, Jeffrey D. Smith, Lisa L. Bergmann, Jakub K. Famulski

**Affiliations:** ^1^Saha Aortic Center, University of Kentucky, Lexington, KY, United States; ^2^Saha Cardiovascular Research Center, University of Kentucky, Lexington, KY, United States; ^3^Department of Family Medicine, University of Kentucky, Lexington, KY, United States; ^4^Department of Surgery, University of Kentucky, Lexington, KY, United States; ^5^Department of Physiology, University of Kentucky, Lexington, KY, United States; ^6^Department Radiology, University of Kentucky, Lexington, KY, United States; ^7^Department of Biology, University of Kentucky, Lexington, KY, United States

**Keywords:** zebrafish, aorta, familial aortopathy, *SMAD3*, pathogenic variant

## Abstract

In human, pathogenic variants in smad3 are one cause of familial aortopathy. We describe a novel *SMAD3* variant of unknown significance (VUS), V244F, in a patient who presented with aortic root dilation, right coronary artery ectasia, abdominal aortic aneurysm, right vertebral artery atresia, and cavernoma. Determination of variant pathogenicity impacted multiple aspects of the patient’s care, including the most appropriate surgical threshold for which to recommend a valve-sparing aortic root replacement. To determine whether the newly identified *SMAD3* variant, and whether *SMAD3* induced aortopathy in general, can be assayed in a zebrafish embryo model, we injected *smad3a* mRNA into Tg[*kdrl*:mCherry] zebrafish embryos. By measuring the size of the dorsal aorta at 48hpf we found a correlation between pathogenic *SMAD3* variants and increased dorsal aortic diameter. The newly identified V244F variant increased dorsal aortic diameter (*p* < 0.0001) similar to that of the pathogenic control variant T261I (*p* < 0.0084). In addition, we examined several previously identified variants of uncertain significance and found P124T (*p* < 0.0467), L296P (*p* < 0.0025) and A349P (*p* < 0.0056) to behave like T261I. These results demonstrate that the zebrafish embryo assay was successful in validating known pathogenic variants, classifying our newly identified variant V244F as likely pathogenic, and classifying previously identified variants P124T, L296P, and A349P as likely pathogenic. Overall, our findings identify a novel *SMAD3* variant that is likely pathogenic as well as offer a new mechanism to model *SMAD3* VUSs *in vivo*.

## Introduction

Aortic aneurysms are permanent dilations that increase an individual’s risk for aortic rupture or dissection, a tear that occurs between layers of the aortic wall. Ruptured aortic aneurysms and dissections are estimated to cause more than 17,000 deaths in the United States each year (1, 2). Aneurysms in the proximal regions of the thoracic aorta have been associated with several genetic mutations. These mutations can cause syndromes (such as Marfan syndrome) or isolated dilation of the thoracic aorta (called Familial Thoracic Aortic Aneurysm and Dissection or FTAAD). FTAAD is believed to account for at least 20 percent of thoracic aortic aneurysms and dissections (3). Gene panels are now available to determine the presence of a FTAAD mutation. However, many mutations in these genes have never been functionally verified and are given the assignment of variants of uncertain significance (VUS). These VUSs could be disease-causing or normal variants that occur in the population. Additional work is needed to determine whether (1) the mutation causes aortic dilation and (2) current pharmacological approaches for aortic aneurysm benefit patients harboring the specific mutation.

Mutations in *SMAD3* cause 2% of heritable aortopathy (4). Mutations in other genes, such as *FBN1* (5), *TGFBR1* (6), *TGFBR2* (7, 8), *MYH11* (9), *ACTA2* (10), *MYLK* (11), *PRKG1* (12) have been identified as a cause of up to another 25% of familial aortopathy (13). These genes encode proteins that are necessary for either the contraction of smooth muscle cells or transduction of transforming growth factor-beta (TGF-β) signaling. SMAD3, specifically, is an essential protein in TGF-beta signaling. TGF-β ligand binding of the type II TGF-β receptor phosphorylates type I receptors in order to activate intracellular SMAD-dependent and SMAD-independent cascades. In SMAD-dependent signaling, SMAD2/SMAD3 become phosphorylated, bind the transcription factor SMAD4, and enter the nucleus to interact with a SBE (Smad Binding Element), modulating the expression of target genes downstream. *SMAD3* has been well characterized to play a functional role in familial aortopathy (14, 15). Although the molecular mechanism by which genetic mutations in SMAD3 and other TGF-beta signaling molecules lead to TAA is unknown (16), Gong et al. found that SMAD3 deficiency could impair differentiation of vascular smooth muscle cells from cardiovascular progenitor cells (CPC-VSMC) in human-induced pluripotent stem cells. Furthermore, SMAD3^–/–^ CPC-VSMC contractility was significantly decreased compared to wild type CPC-VSMC (16). Loss of SMAD3 function also increased collagen fiber deposition and enhanced stiffness in CPC-VSMC tissue rings, consistent with the clinical phenotype seen in people with loss of function mutations in *SMAD3* (16). Currently, over 65 mutations have been discovered in people with FTAAD, however, many of these mutations, up to 35, are classified as variants of uncertain significance (VUS) (14). Additional work needs to determine whether these 35 mutations cause FTAAD in these patients or if they are benign variants.

In addition to aortic aneurysms, *SMAD3* mutations can cause a number of additional vascular manifestations, including intracranial aneurysm, abdominal aortic aneurysm, iliac artery aneurysm, and arterial tortuosity (4). Cardiac manifestations include mitral valve prolapse, myxomatous valve disease, and atrial fibrillation. Extravascular manifestations include early onset osteoarthritis as well as degenerative disk disease (4). Although inheritance occurs in an autosomal dominant fashion, reduced penetrance and phenotypic variability exists within families (4).

In our current study, we identify a novel VUS in *SMAD3*, V244F, in a patient presenting with aortic root aneurysm, right coronary artery ectasia, abdominal aortic aneurysm, right vertebral artery atresia, and cavernoma. Definitive classification of the variant had many implications for patient care. First, if pathogenic, the patient is an immediate candidate for a valve-sparing aortic root replacement. This is because surgery would generally be offered to a person at an aortic root diameter of 50-55 mm. However, in the setting of a pathogenic *SMAD3* variant, the threshold to consider surgical repair is 4.5 cm (17). Second, classification can affect pharmacologic management, such as the initiation of an angiotensin-receptor blocker (17). Finally, a pathogenic classification helps direct screening and surveillance strategies for the patient’s three children. To validate the pathogenicity of this mutation, and therefore offer potential therapeutic options to the patient, we designed a zebrafish embryo based *in vivo* assay. Using a transgenic line Tg[*kdrl*:mCherry] to visualize vasculature, we tested known pathogenic variants of *SMAD3* as well as our newly identified VUS. Results from this novel assay indicate that SMAD3^*V*244*F*^ acts like a pathogenic variant of *SMAD3* and that zebrafish modeling of FTAAD associated *SMAD3* variants may be possible.

## Results

**FIGURE 1:**
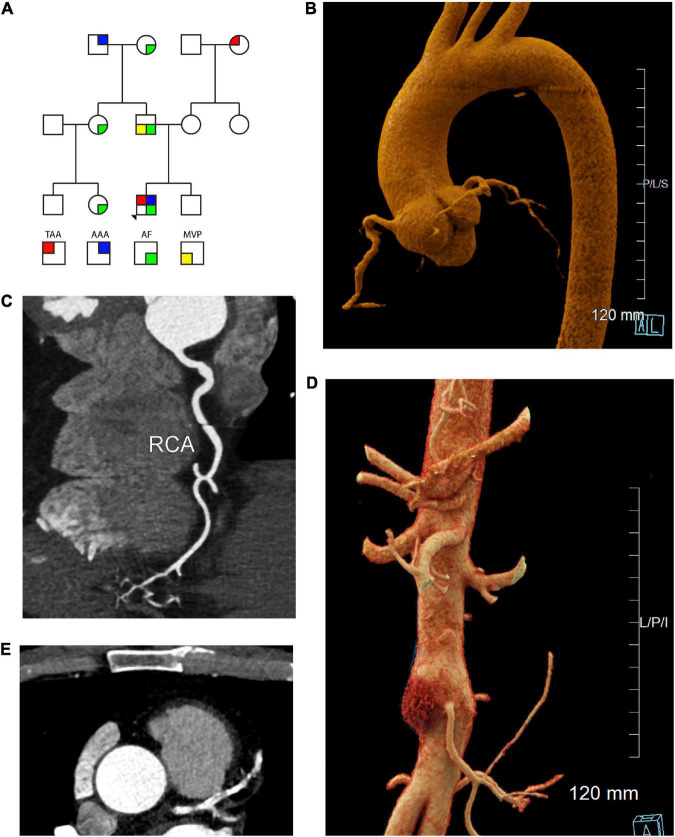
Cardiac, aortic, and coronary artery findings in a 55 year old male with the *SMAD3* V244F variant. **(A)** Pedigree demonstrating sex and phenotype. Squares and circles represent males and females, respectively. TAA is red upper left quadrant. AAA is blue upper right quadrant. AF is green lower right quadrant. MVP is yellow lower left quadrant. **(B)** Aortic root aneurysm and Focal bilobed fusiform ectasia of the proximal RCA. Linear reformat of coronary CTA was created using TeraRecon (Durham, North Carolina). **(C)** Axial CTA demonstrating tortuosity and ectasia of the right coronary artery (RCA). **(D)** Infrarenal abdominal aortic aneurysm, 37 mm in maximum diameter. Mural hematoma in the right lateral aspect of the aneurysm is displayed in red. Cinematic 3D reformatted images of CTA abdomen and pelvis. Cinematic 3D were created using *syngo*.via (Siemens, Erlangen). **(E)** Calcification of the left coronary artery and its branches with 25-49% stenosis in the LAD. TAA is thoracic aorta aneurysm, AAA is abdominal aortic aneurysm, AF is atrial fibrillation, MVP is mitral valve prolapse 3D is 3-dimensional, CTA is computed tomographic angiography, RCA is right coronary artery, LAD is left anterior descending artery.

A 55 year-old male with a past medical history of paroxysmal atrial fibrillation, hypertension, and thoracic aortic aneurysm presented for evaluation of heritable aortopathy. Past history included eosinophilic esophagitis as well as atraumatic herniated disc at the age of 49. Family history was notable for fatal aortic dissection in a maternal grandmother in the eighth decade of life, abdominal aortic aneurysm in a paternal grandfather in the eighth decade of life, as well as atrial fibrillation in his father, paternal grandmother, paternal aunt, and paternal cousin (Figure 1A). Patient was a non-smoker. Aortopathy panel testing was performed by Invitae, which included sequencing of the following genes: *ACTA2, CBS, COL3A1, COL5A2, EFEMP2, FBN1, FBN2, FLNA, MAT2A, MED12, MYH11, MYLK, NOTCH1, PLOD1, PRKG1, SKI, SCL2A10, SMAD3, SMAD4, SMAD6, TGFB2, TGFB3, TGFBR1, and TGFBR2*. No pathogenic variants were identified. One variant of uncertain significance was identified in *SMAD3*, c.730G > T (p.Val244Phe). Segregation analysis in the patient’s parents and brother was recommended, but family members were not willing to undergo testing at the time. Given the young age of the patient’s three children, segregation analysis in progeny was deferred pending variant reclassification. At the time of initial evaluation, computed tomographic angiography (CTA) of the chest demonstrated an aortic root aneurysm of 45 mm (Figure 1B). The right coronary artery was found to be focally ectatic (>6.0 mm) (Figure 1C). Computed tomography (CT) of the abdomen and pelvis did not reveal aneurysmal disease. Screening of head and neck vasculature was deferred by the patient. Three years later, surveillance CTA of the chest revealed a stable aortic root diameter. However, CTA of the abdomen and pelvis revealed interval development of an infrarenal aortic aneurysm with mural atherosclerotic plaque, which had increased in size from a diameter of 25 mm x 25 mm to 32 mm x 37 mm (Figure 1D). Due to onset of intermittent chest discomfort with exertion, cardiac CT was performed. Cardiac CT revealed severe coronary calcification (Figure 1E) with an Agatston score of 383 using the AJ-130 method, which represents the 91st percentile when matched for age, gender, and ethnicity. In the AJ-130 method, coronary artery calcific densities of at least 130 Hounsfield units (HU) with an area of at least 1 mm^2^ are scored 1 to 4, and each score is multiplied by the area. Based on these results, vascular age was calculated to be 82 years. The proximal to mid left anterior descending coronary artery (LAD) and proximal right coronary artery (RCA) contained mixed plaque causing mild stenosis. Cardiovascular risk factors were optimized, including initiation of high-intensity statin. Regular echocardiography monitoring revealed an absence of mitral valve prolapse or significant valvular regurgitation. After six months, aortoiliac duplex demonstrated stable size of the infrarenal abdominal aortic aneurysm (AAA).

**FIGURE 2:**
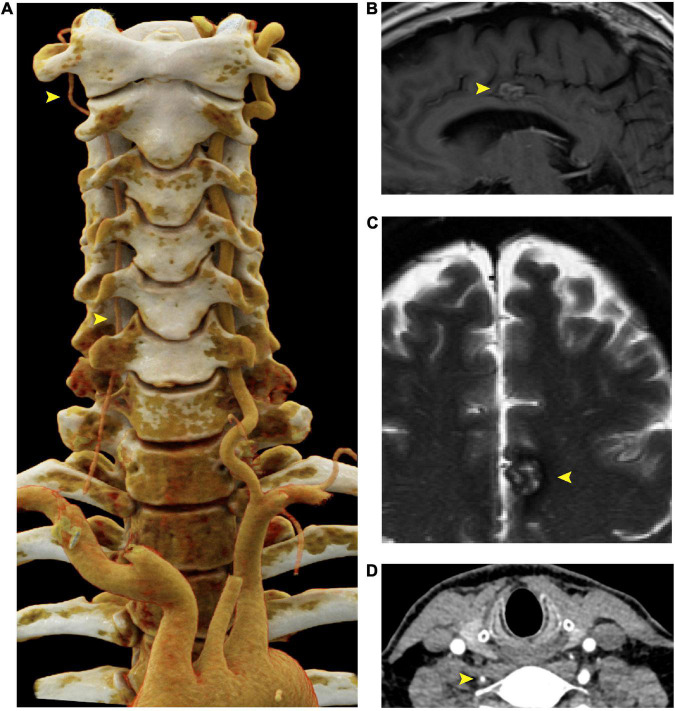
Right vertebral artery atresia and cavernoma in a 55 year old male harboring a *SMAD3* V244F variant. **(A)** Cinematic 3D reformatted images of the atretic < 2mm right vertebral artery. The origin of the right vertebral artery was sufficiently narrow in caliber that volume averaging resulted in below-threshold density required for visualization in the reformat. For best visualization of the vertebral arteries, the carotid arteries were digitally removed using *syngo*.via (Siemens, Erlangen). The dominant left vertebral artery measures 5 mm in diameter throughout most of its length, which is the upper limit of normal. **(B)** Sagittal T1-weighted FLAIR MRI of the brain at the midline shows a cavernous malformation in the left mid cingulate gyrus, just superior to the body of the corpus callosum. **(C)** Axial T2-weighted MRI with fat saturation demonstrates characteristic signal intensity of cavernous malformation with central hyperintensity, peripheral hypointensity, and susceptibility artifact. **(D)** Axial CTA demonstrating atretic < 2mm right vertebral artery. FLAIR is fluid-attenuated inversion recovery, MRI is magnetic resonance imaging.

Screening CTA of the head and neck revealed atresia of the right vertebral artery (Figure 2A), but no evidence of intracranial aneurysm. Six months later, the patient underwent magnetic resonance angiography (MRA) of the head, neck, chest, abdomen, and pelvis, which revealed stability of both the thoracic aorta aneurysm (TAA) and AAA as well as mild tortuosity of the external iliac arteries. Incidental findings included a 1.7 cm cavernous malformation in the left cingulate gyrus (Figures 2C, D), which remained stable over the course of six months and continues to be monitored.

Sequencing revealed a change in the sequence of exon 6 in *SMAD3* resulting in a change of valine with phenylalanine at codon 244. The valine residue is highly conserved and there is a small physicochemical difference between valine and phenylalanine. The variant was not present in population databases and *in silico* analyses did not agree on the potential impact of the missense change (SIFT: “Deleterious”; PolyPhen-2: “Probably Damaging”; Align-GVGD: “Class C0”). However, classification of the variant as pathogenic would alter the patient’s medical care in multiple ways. First, patients with pathogenic mutations in *SMAD3* are recommended to have aortic root replacement at smaller diameters compared to the general population (17, 18). Given that segregation analysis in the extended family was not an option at the time, a project to investigate the feasibility of modeling *SMAD3* mutations in zebrafish was undertaken.

**FIGURE 3:**
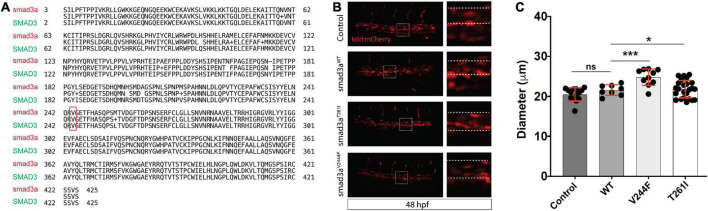
Zebrafish embryo-based assay for *SMAD3* variant pathogenesis. **(A)** Degree of conservation in amino acid sequence between Smad3a in zebrafish and human SMAD3. Overall, similarity is at ∼97% indicating a very high level of conservation between the species. Residue V244 is highlighted to emphasize total conservation between zebrafish and human Smad3. **(B)** Confocal images of the tail aorta/vein labeled in Tg[kdrl:mCherry] in 48hpf embryos. White dashed boxes indicate the enlargement regions used to highlight differences in diameter (white dashed lines). Embryos imaged were either control, injected with smad3a^WT^ mRNA or smad3a^T261I^ or smad3a^V244F^. **(C)** Quantification of diameter counts. Each point represents an individual embryo measured. Standard deviation is depicted in red. *p < 0.05, ***p < 0.001.

**FIGURE 4:**
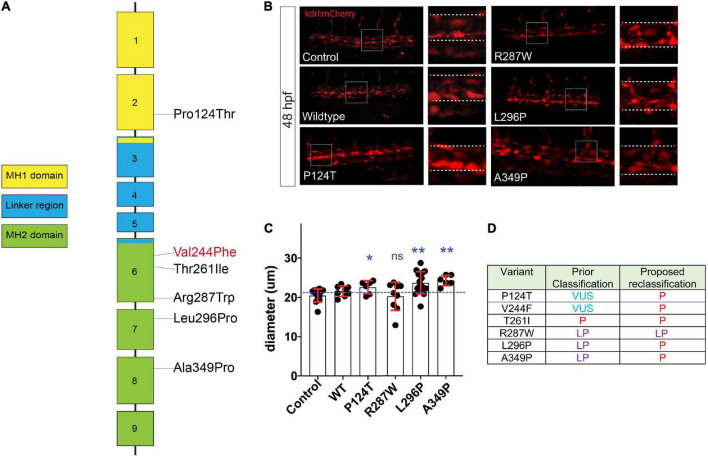
Assessment of *SMAD3* VUS pathogenicity using a zebrafish embryonic assay. **(A)** Diagram of the spatial distribution of specific VUS mutations examined. **(B)** Confocal images of the tail aorta/vein labeled in Tg[kdrl:mCherry] in 48hpf embryos. White dashed boxes indicate the enlargement regions used to highlight differences in diameter (white dashed lines). Embryos imaged were either control, injected with smad3a^WT^ mRNA or smad3a^P124T^, smad3a^R287W^, smad3a^L296P^ or smad3a^A349P^. **(C)** Quantification of diameter counts. Each point represents an individual embryo measured. Standard deviation is depicted in red. Variants were compared to WT: *p < 0.05, **p < 0.01. **(D)** Summary of VUS classification changes based on the zebrafish embryonic assay results. P, pathogenic, LP, likely pathogenic.

To model and assess potential pathogenicity of the SMAD3^*V*244*F*^ variant we employed the zebrafish embryo system. Zebrafish embryos are readily accessible for manipulation, including mRNA injections. To that end we sought to determine if overexpression of SMAD3 pathogenic variants, possibly including SMAD3^*V*244*F*^ would have functional consequences on developing vasculature. Alterations in vasculature integrity or assembly would be considered as validations variant dysfunction when compared to wildtype. As such, we employed a transgenic line of zebrafish which labels the developing vasculature, Tg[*kdrl*:mCherry]. Human and zebrafish smad3a share 97% identify and total conservation of V244 (Figure 3A). First, we generated the T261I variant of smad3a which has been shown to be pathogenic (15) as our positive control. Zebrafish smad3a cDNA was cloned into the pCS2 vector and subsequent site directed mutagenesis was performed to generate the zebrafish T261I and V244F variants. Using an *in vitro* transcription system, we next generated mRNA for WT, T261I and V244F *smad3a*. 1 cell stage embryos were injected with 50pg of WT, V244F or T261I mRNA and allowed to develop up to 48hpf. At this stage of development, the dorsal vein and aorta are lined with endothelial cells expressing the mCherry fluorescent protein. Using 3D confocal microscopy, we captured images of the vasculature at the termination of the yolk extension and subsequently compared the diameters between treatments (Figure 3B). In doing so, we observed that injection of WT (*n* = 8) smad3a mRNA had no significant effect when compared to un-injected controls (*n* = 14). When we injected our smad3a pathogenic positive control mRNA, T261I, we saw an increase in the diameter (6.3%, *p* < 0.0068, *n* = 9), suggesting our assay can correlate pathogenic smad3 variants with an abnormal vasculature phenotype. Importantly, when we injected V244F mRNA we again saw an increase in diameter measurements of 21% (*p* < 0.0001, *n* = 10) (Figure 3C). One-way ANOVA analysis of the variants, WT and controls resulted in p value of 0.001. Taken together we concluded that our zebrafish embryo assay can correlate SMAD3 pathogenicity to alterations of vasculature at early developmental time points and thus classify V244F as likely pathogenic. Considering the results, we sought to further expand our assay by examining several other SMAD3 variants previously classified as VUS or likely pathogenic. These include P124T (19), R287W (15, 20), L296P (20) and A349P (21). All four are conserved between human and zebrafish smad3a. Overall, the variants chosen span the MH1 and MH2 domains of Smad3 (Figure 4A). Upon injection of corresponding mRNAs we again collected measurements and observed an increase in diameter for P124T (10%, *p* < 0.0467, *n* = 6), L296P (15.5%, *p* < 0.0025, *n* = 17) and A349P (18.3%, *p* < 0.0056, *n* = 6) (Figures 4B, C). One-way ANOVA analysis of the variants, WT and controls, resulted in p value of 0.001. Thus, these VUSs are also classified pathogenic according to our assay. R278W did not differ from controls (*n* = 9) and therefore is either not pathogenic or remains a likely pathogenic (LP) VUS (Figure 4D). Taken together, our zebrafish embryo assay was successful in validating known pathogenic variants and classifying our newly identified variant V244F as well as previously identified variants P124T, L296P, and A349P as likely pathogenic.

## Materials and methods

### Zebrafish care

Zebrafish were maintained using husbandry procedures approved by University of Kentucky IACUC committee (protocol #2021-3781). Embryos were kept at 28.5°C in E3 embryo media. Tg[*kdrl*:mCherry] transgenic line was used to visualize vasculature (22).

### mRNA synthesis and injections

Coding sequence for zebrafish *smad3a* (zfsmad3) was amplified using 24hpf cDNA and cloned into pCS2 using BamH1 and *Xho*I. For variant synthesis, overlapping primers with corresponding sequence changes were used to amplify pCS2-zfsmad3 with Fusion taq polymerase. Primer sequences can be found in Supplementary Figure 1. Template plasmid was removed via Dpn1 digest and mutagenized product transformed into DH5a cells (NEB). Sanger sequencing (Eurofins genomics) was used to confirm the mutations using ClustalW alignment with wildtype *smad3a* coding sequence using MacVector software (Supplementary Figure 1). WT or variant *smad3a* mRNA was synthesized using the message Machine SP6 kit (Ambion) and purified using Sigma Spin clean up columns. Injections were done using 1 cell stage embryos, 50 pg of mRNA was injected into the cell using a pressure microinjector along with dextran green as a marker for injection control. At minimum 25 embryos were injected for each mRNA construct. At 24 hpf the embryos were screened for green fluorescence using a stereo microscope. Positive embryos were subsequently grown to 48hpf and fixed in 4% PFA.

### Confocal imaging and measurements

Whole zebrafish embryos were mounted in low melting point agarose (sigma) on glass cover slip bottom Fluorodishes (WPI). Images were collected using a Nikon C2 + confocal microscope using a 20 × 0.95 NA objective. 3D stacks were collected using 3 μm steps and analyzed using Nikon Elements software. Diameter measurements were done using Nikon Elements software. Statistical significance was calculated using one-way ANOVA with Bonferroni’s multiple comparisons test for individual comparisons using Prism software.

## Discussion

This study advances current knowledge in multiple ways. First, we identified a novel variant in *SMAD3*, c.730G > T (p. Val244Phe) and provided clinical and laboratory data to support its classification as likely pathogenic. This discovery can have immediate clinical relevance for patients. For the patient described in this paper, he would be considered a candidate for valve-sparing aortic root surgery as his aortic root was above the 4.5 cm threshold. Furthermore, we initiated an angiotensin receptor-blocker, which generally would not have been considered in the absence of hypertension if the patient was not suspected to have SMAD3-associated aortopathy. In addition to advancing knowledge to inform this specific patient’s care, we also demonstrated the feasibility of modeling *SMAD3* variants using a zebrafish embryo system. In fact, we were able to take previously-identified variants of uncertain significance in *SMAD3* (P124T, L296P, and A349P) and provide data from a functional assay to assist in their reclassification as likely pathogenic. In our interpretation, the R287W variant remains suspicious due to the large range in diameter measurements. We suspect that increasing the number of embryos testing for R287W pathogenicity may result in reclassification of this variant as well.

Limitations of the study include a lack of regionally-specific anatomic correlation between the zebrafish dorsal aorta and the regions known as the ascending thoracic aorta, descending thoracic aorta, and abdominal aorta in human. It is important to note that at the time aortic diameter measurements were made in the zebrafish, the primordial heart had not yet formed. Our measurements were taken at the end of the yolk extension, in the region of the intersegmental vessels, downstream from the area where the lateral dorsal aortas have joined together to form a single blood vessel. A portion of the dorsal aorta develops into the zebrafish heart (23, 24). Therefore, it is very difficult to say exactly which portion of the primordial zebrafish aorta is the most similar to the human ascending aorta. We feel that this is less of a concern when discussing modeling *SMAD3* variants in a zebrafish embryo system because, per the patient case described above, *SMAD3*-dependent aortic pathology is not limited to only the ascending thoracic aorta in humans. The patient in whom this *SMAD3* variant was first identified has developed both a thoracic aortic aneurysm as well as an abdominal aortic aneurysm. To this extent, we believed that it was most critical that we measured aortic diameter in the exact same location for all the zebrafish in our study. Whether or not that region can be exactly correlated with the ascending aorta in humans was thought to be less critical.

An additional limitation of the study includes the inability to determine how variants impact vascular formation in later stages of development. Given that mRNAs are injected at the single cell embryonic stage, we limited aortic diameter measurements prior to the timeframe in which we would have expected the mRNA to remain active. 48-72 hpf are the expected maximum timelines for durability of injected mRNAs. Future mutagenesis of the germline, such as those that can be done more easily with the widespread availability of CRISPR-Cas9-mediated homologous recombination techniques, will enable observation of variant impacts at later stages of development. Another limitation of the use of mRNA injection includes the inability to model all types of variants identified, especially those that result in frameshift and nonsense mutations as these are most likely not dominant. We therefore recognize that certain *SMAD3* variants are not amenable to a mRNA injection approach.

One of the most compelling reason to pursue the embryonic zebrafish system for modeling of *SMAD3* variants is the potential for high-throughput as well as modeling of pharmacologic efficacy. The amount of time and money to conduct this study is minimal compared to the time and money that would be required to model this variant in mouse. Even when money is available, patient care can require more immediate decision-making. Secondly, an advantage of this model is that zebrafish can absorb pharmacotherapeutics dissolved in their ambient water while they swim (25). This can be an efficient and inexpensive way to model pharmacologic impacts compared to other alternatives that enable readouts at the system level of physiology. Furthermore, zebrafish skin is translucent, enabling detection of changes in vascular phenotype over time without requiring echocardiology or computed tomography/magnetic resonance imaging. Additional studies could provide data to demonstrate the promise of certain pharmacologic interventions in humans, both with *SMAD3*-induced aortopathy as well as those with mutations in additional genes that cause familial aortic aneurysms.

## Data availability statement

The original contributions presented in this study are included in this article/Supplementary material, further inquiries can be directed to the corresponding authors.

## Ethics statement

The animal study was reviewed and approved by the University of Kentucky Institutional Animal Care and Use Committee.

## Author contributions

MS: study conception and design, data interpretation, writing and editing the manuscript. JS and LB: figure preparation, reviewing and editing the manuscript. JF: study conception and design, zebrafish manipulation, microscopy, data analysis and interpretation, and writing and editing. All authors contributed to the article and approved the submitted version.
