# Supplementary figures and images for: Novel SMAD3 variant identified in a patient with familial aortopathy modeled using a zebrafish embryo assay
Source: Front Cardiovasc Med. 2023 Feb 28;10:1103784. doi: 10.3389/fcvm.2023.1103784 (PMC10011127; doi:10.3389/fcvm.2023.1103784)

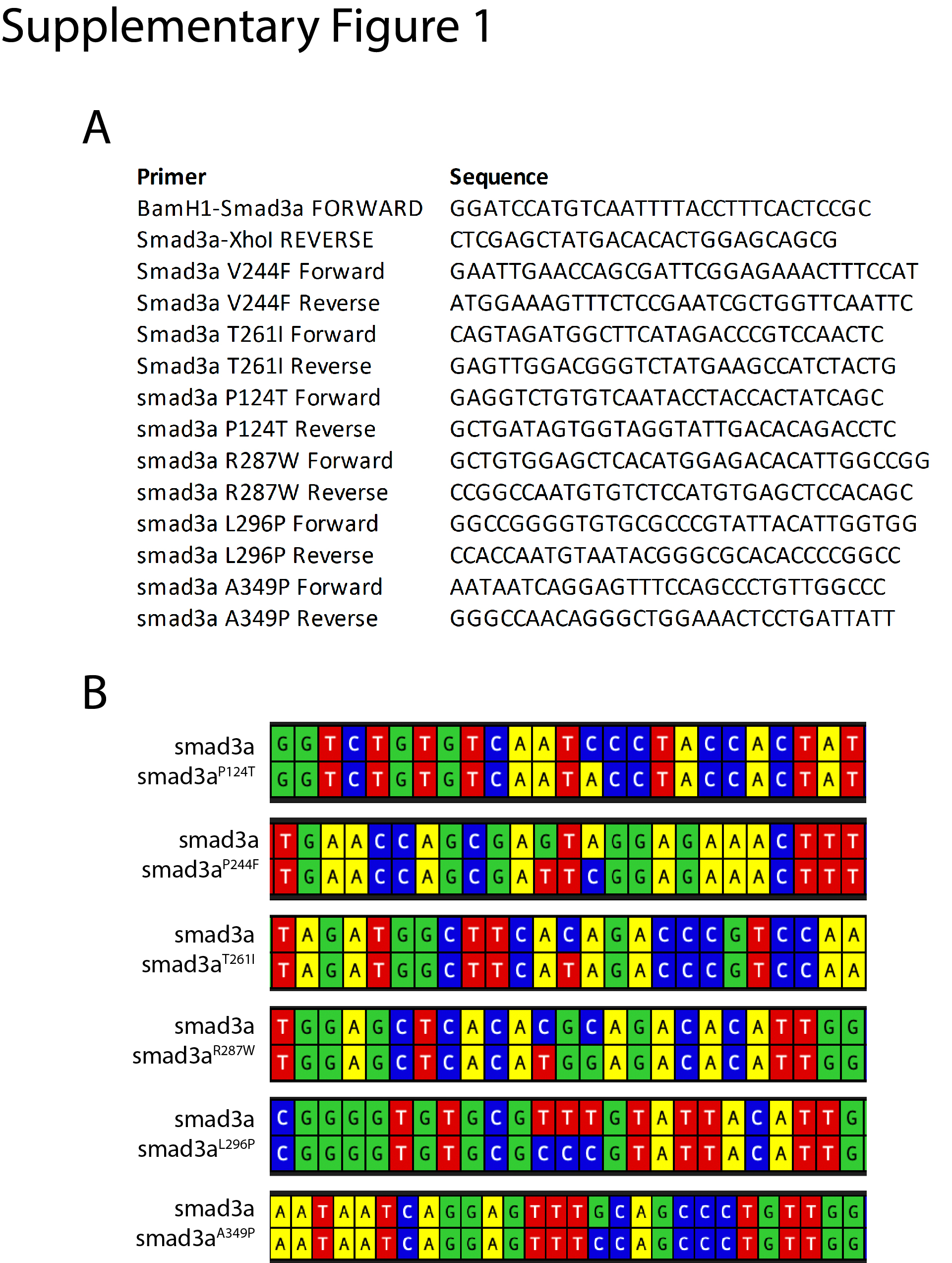

Supplement: Supplementary file 1 [file Image_1.JPEG]
